# The impact of COVID-19-related restrictions on pregnancy and abortion rates in the Republic of Georgia

**DOI:** 10.1186/s12913-023-10417-7

**Published:** 2023-12-18

**Authors:** Nia Khachidze, Tinatin Manjavidze, Erik Eik Anda, Ingvild Hersoug Nedberg, Ingvild Fossgard Sandøy, Charlotta Rylander

**Affiliations:** 1 Department of Community Medicine, Faculty of Health Sciences, UiT The Arctic University of Norway, Tromsø, Norway; 2 Department of Health and Care Sciences, Faculty of Health Sciences, UiT The Arctic University of Norway, Tromsø, Norway; 3 Center for Intervention Science in Maternal and Child Health, Centre for International Health, Department of Global Public Health and Primary Care, University of Bergen, Bergen, Norway

**Keywords:** COVID-19, Restrictions, Lockdown, Pregnancy rate, Abortion rate, Birth registry

## Abstract

**Background:**

The Republic of Georgia implemented COVID-19-related restrictions starting on 31 March 2020, when it imposed a 1-month strict lockdown, after which the country continued with some form of restrictions for 1 year. These restrictions created barriers to healthcare access, affected healthcare services, caused severe economic degradation, and changed reproductive behavior. The aim of this study was to explore the impact of COVID-19-related restrictions on pregnancy and abortion rates in Georgia.

**Methods:**

Information on pregnancy, abortion, and related variables was extracted from the Georgian Birth Registry from January 2018 through April 2022. The final study sample included 232,594 pregnancies and 86,729 abortions. We used interrupted time series analysis to study the impact of COVID-19-related restrictions.

**Results:**

There were slightly decreasing trends in pregnancy and abortion rates in the pre-pandemic period (1 January 2018-31 March 2020). During the 1-month strict lockdown (1 April-30 April 2020), pregnancy and abortion rates decreased in all investigated age groups. There were no substantial differences in pregnancy or abortion rates in the pandemic period (for pregnancies: 1 April 2020-30 June 2021; for abortions: 1 April 2020-30 April 2022) compared to the pre-pandemic period. The precision of all estimates suggested that both small increases and decreases in pregnancy and abortion rates are reasonably compatible with our data.

**Conclusions:**

Despite the 1-year-long COVID-19-related restrictions, our results did not indicate substantial long-term changes in pregnancy or abortion rates during the study period for any age group. This may indicate that the restrictions did not substantially influence access to contraception, abortion services, or reproductive behavior.

**Supplementary Information:**

The online version contains supplementary material available at 10.1186/s12913-023-10417-7.

## Introduction

The COVID-19 pandemic has had a significant impact on our societies. To prevent virus transmission, more than two-thirds of countries implemented lockdowns and strict social distancing measures. This caused major disruption to healthcare systems, and especially to sexual and reproductive health (SRH) care services [1–4]. Increased barriers to family planning and safe abortion services during the pandemic have been commonly reported [5], and border restrictions impacted the supply of modern contraception [3, 6]. The Guttmacher Institute predicted that the pandemic could cause a 10% drop in SRH care utilization in low- and middle-income countries (LMICs), and estimated that this would result in approximately 49 million additional women with unmet contraceptive needs, 15 million unintended pregnancies, and 3 million unsafe abortions [7]. Adolescents represent an especially vulnerable group that was impacted by the pandemic in terms of access to SRH care [8, 9]. Indeed, young people faced obstacles even after many countries adopted telemedicine services during the pandemic [10], because some jurisdictions have age restrictions on access to contraception via telemedicine services [11]. Apart from its impact on healthcare services, the pandemic also influenced people’s social lives, mental health, and socio-economic conditions, which may have affected conception rates, abortion rates, and birth rates, as factors like financial instability can change reproductive intentions and behavior [12, 13].

Due to major differences in the availability of contraception and in the educational levels of women across countries, COVID-19-related restrictions may have had different effects in LMICs than in high-income countries (HICs) [13–15]. Indeed, challenges in accessing contraception during the pandemic may have resulted in an increase in unplanned pregnancies in many LMICs [16], while studies from HICs have observed declining birth rates and decisions to delay pregnancies [13, 15].

The first case of COVID-19 in the Republic of Georgia was recorded on 26 February 2020, but Georgia had already established an Interagency Coordination Council and enforced a mandatory quarantine for certain travelers in January 2020. Georgia implemented COVID-19-related restrictions starting on 31 March 2020, when it imposed a strict lockdown: it closed all its educational and cultural institutions, banned international travel, declared a state of emergency, and imposed a curfew. At the same time, the country gradually decentralized laboratory services and improved geographical access to COVID-19 testing [17], and the Ministry of Internally Displaced Persons from Occupied Territories, Labor, Health and Social Affairs of Georgia announced recommendations regarding the temporary suspension of non-emergency health services, which might have negatively affected SRH care services.

The strict lockdown lasted for 1 month; the government started to lift restrictions in an incremental manner starting at the end of April 2020. Some restrictions were still in place during the subsequent summer tourist season, when Georgia experienced a surge in cases that peaked in December 2020 with a 7-day average incidence of 120.4 per 100,000. As of 15 December 2020, Georgia had the highest incidence of COVID-19 among European countries [17], and the hospital sector was under pressure because of the high number of hospitalized patients. These developments forced the government to reinstate some of the previously lifted COVID-19-related restrictions, including imposing a public transportation ban, remote work and education, and a curfew. Some of these restrictions were lifted again in the first quarter of 2021, but the curfew lasted until July 2021. A third, rather small wave of COVID-19 occurred in May 2021, followed by another wave in August of the same year [17]. The biggest wave occurred between December 2021 and February 2022, when almost no restrictions remained in the country [17].

In addition to stress caused by the uncontrolled spread of the virus, the country faced significant economic declines following job losses that severely affected the tourism, entertainment, and recreation sectors. The unemployment rate increased from 17.6% in 2019 to 18.5% in 2020 and peaked at 20.6% in 2021. The highest unemployment rate was observed in the age group 15–24 years, increasing from 27.8% in 2019 to 46.5% in 2021 [18]. All the above-mentioned factors could have affected access to contraception and abortion services, as well as reproductive behaviors. We hypothesize that these factors would have affected adolescents the most. Hence, this paper aimed to explore the impact of COVID-19-related restrictions on pregnancy and abortion rates in Georgia using data from the Georgian Birth Registry (GBR).

## Materials and methods

### The Georgian Birth Registry

In 2016, Georgia launched the nationwide GBR. Every clinic that provides antenatal care, deliveries, postpartum care, and abortions is obliged by law to submit digital information to the GBR on mothers and/or newborns before they are discharged or transferred to another healthcare facility. Moreover, the state health program in Georgia finances perinatal services, but it does not provide coverage for abortion services. Pregnant women are registered in the GBR at their first contact with any of these obstetric services; however, the GBR does not contain information on home births (around 0.2% of all births in the country) [19] or abortions performed with over-the-counter drugs, which are accessible without a prescription in Georgia. There are more than 500 variables in the GBR, including information on maternal and paternal characteristics, maternal pregnancy history, current pregnancy, the intrapartum period, and newborns. Delivery/abortion date and gestational age were used to estimate the conception date of each pregnancy. Detailed information on the implementation of the GBR is described elsewhere [20]. The number of births and deaths in the GBR are systematically validated against the Vital Registration System, and newborn coverage in the registry has consistently been above 99% since 2017 [21].

### Study sample

The initial study sample consisted of two main groups: “pregnancies”, which consisted of pregnancies registered in the GBR with a conception date between 1 January 2018 and 30 June 2021 and a recorded delivery or abortion date; and “abortions”, which consisted of induced or spontaneous abortions that occurred between 1 January 2018 and 30 April 2022. Women who do not seek obstetric services until they go into labor (~ 5.3%) [22] are not registered in the GBR until delivery. Therefore, we chose to cut-off inclusion for pregnancies at 30 June 2021, as conceptions after this date could not arrive to full term before 30 April 2022, which was the latest available update from the GBR.

**Fig. 1:**
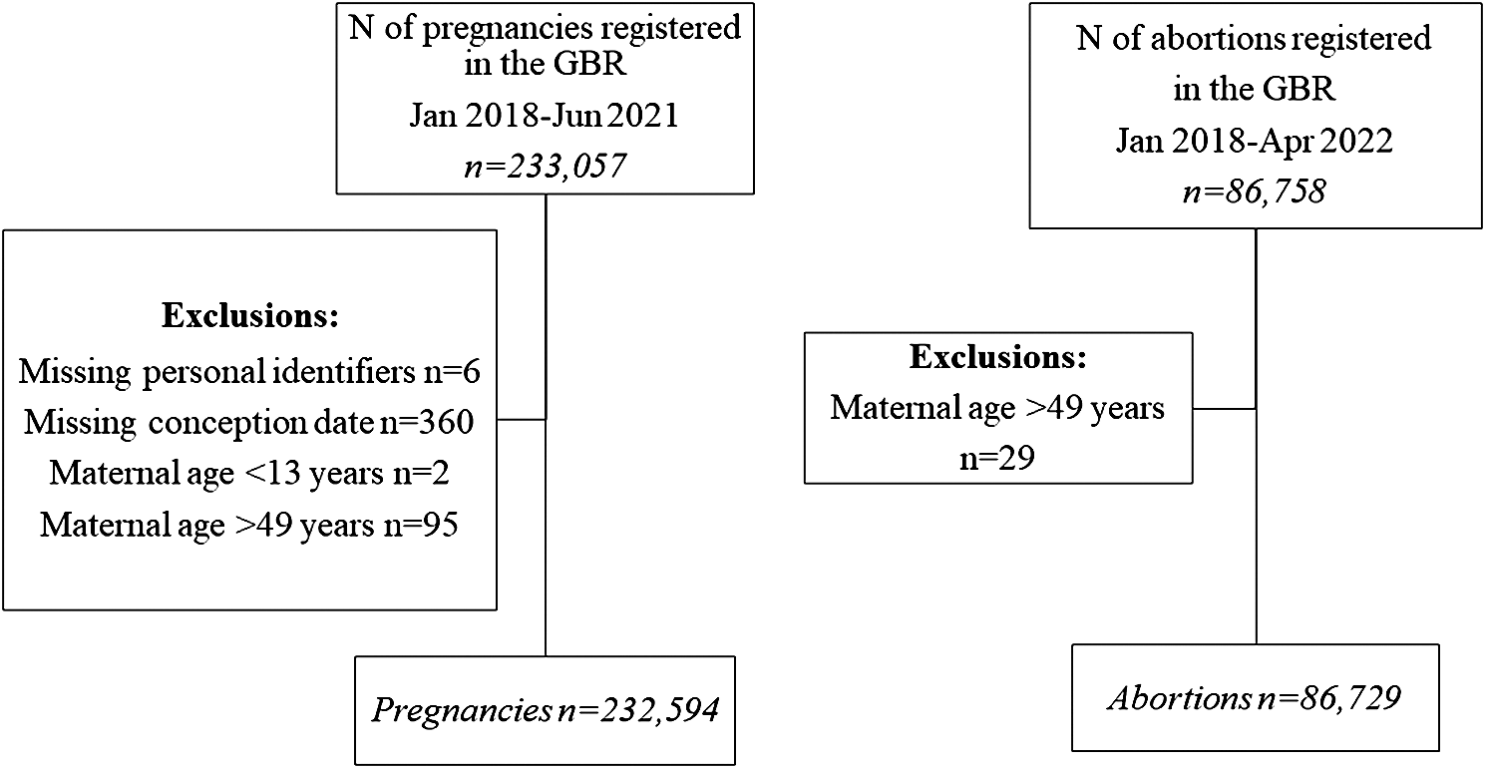
Flow chart of pregnancies and abortions included in the study sample

The initial pregnancies group consisted of 233,057 pregnancies. Mothers with missing personal identifiers (n = 6), pregnancies for which a conception date could not be calculated (missing conception date) (n = 360), as well as biologically implausible values of maternal age (< 13 years; n = 2; >49 years; n = 95) were excluded, leaving 232,594 pregnancies in the final sample (Fig. 1). The initial abortions group consisted of 86,758 abortions; after excluding biologically implausible values of maternal age (> 49 years; n = 29), the final sample consisted of 86,729 abortions.

### Variables

Information on maternal age (adolescents: 13–19 years; women: 20–49 years, as well as 5-year age groups 20–24, 25–29, 30–34, 35–39 and 40–49 years), nationality (Georgian, Azerbaijani, Armenian, other, missing), education (primary, secondary, higher, unknown), residency (urban, rural, missing), method of abortion (medical, surgical, missing), date of delivery or abortion, and gestational age at birth or abortion (in weeks) was extracted from the GBR.

The intervention of interest was defined as the 1-year-long COVID-19 related restrictions that began on 31 March 2020. Outcomes were defined as pregnancy and abortion rates in all women of reproductive age (aged 13–49 years), and in adolescents 13–19 years and women 20–49 years separately.

### Statistical analysis

Descriptive statistics for maternal characteristics are presented as frequencies and percentages and gestational age at abortion as mean values with standard deviations. To visualize trends in live births in Georgia from 1994 to 2021, data on absolute number of live births each year was extracted from the National Statistical Service of Georgia and presented as a line diagram.

We used interrupted time series analysis (ITSA) to study the impact of COVID-19-related restrictions on pregnancy and abortion rates, which were calculated for all women, and for adolescents 13–19 years and women 20–49 years separately, as the number of registered pregnancies/abortions per month in each age group divided by the total number of women in each age group per year extracted from the National Statistical Service of Georgia [23].

We calculated baseline rates (January 2018), the monthly rate change in the pre-pandemic period (1 January 2018-31 March 2020), the immediate rate change during the 1-month strict lockdown (1 April-30 April 2020), the monthly rate change in the pandemic period (for pregnancies: 1 April 2020-30 June 2021; for abortions: 1 April 2020-30 April 2022), and the monthly difference in rate in the pandemic period relative to the pre-pandemic period. Single-group ITSAs were performed for all rates of interest for all women, and for adolescents 13–19 years and women 20–49 years separately. Multi-group ITSA analysis was performed to compare pregnancy and abortion rates in adolescents 13–19 years and women 20–49 years. To explore potential heterogeneity in conception and abortion rates across ages in women 20–49 years, we performed additional single-group ITSAs per 5-year age groups as a sensitivity analyses.

The ITSA command performs analyses using the ordinary least-squares regression-based approach. The Newey-West estimator was applied to address autocorrelation. The ITSA method assumes that the pre-pandemic trend would continue into the pandemic period if the intervention of interest (the initiation of COVID-19 related restrictions) had not occurred. As potential confounding factors are likely to change slowly over time, the assumption is that rapid changes related to the intervention of interest will be distinguishable from changes explained by confounding factors if no other interventions occurred in the same time period.

Statistical analyses were performed using Stata version 17.0 (Stata Corporation, College Station, TX, USA) using the ITSA package [24].

## Results

**Fig. 2:**
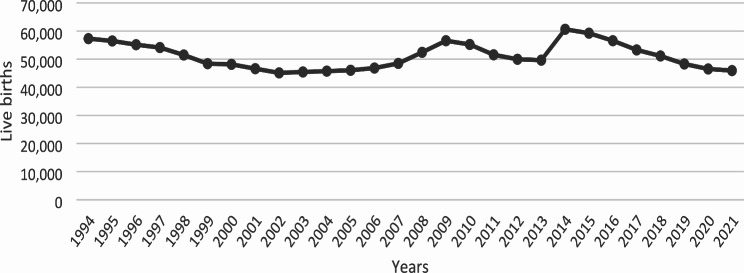
Live births in the Republic of Georgia, 1994–2021 [25]

From 1994 until 2006, the absolute number of live births in Georgia declined steadily (Fig. 2). From 2006 to 2009 and from 2013 to 2014, Georgia experienced a substantial increase in the number of live births, followed by declines in subsequent years.

The majority of pregnancies and abortions during the study period occurred among women 20–49 years, who were of Georgian nationality, with a secondary education, and who lived in urban areas (Table 1). There was only a slight change in the proportion of pregnancies and abortions that occurred among women 20–49 years and adolescents 13–19 years (women 20–49 years: 95.6% vs. 95.1% for pregnancies; 97.4% vs. 97.0% for abortions, adolescents: 4.4% vs. 4.9% for pregnancies; 2.6% vs. 3.0% for abortions) in the pandemic period compared to the pre-pandemic period. The increase in the proportion of women of Georgian nationality during the pandemic period likely reflects the substantial reduction in missing values during that period since it became mandatory to enter information about nationality into the GBR in 2019. Education and residency in the pregnancies and abortions groups varied little between the pre-pandemic and the pandemic periods.

Medical abortion was the most common type of abortion throughout the study period; it increased by 5.3% points in the pandemic period compared to the pre-pandemic period, while surgical abortions declined by 4.4% points. Mean gestational age at abortion remained stable in both periods at around 7 weeks.

**Table 1:** Maternal characteristics of pregnancies and abortions in women of reproductive age (13–49 years)

Variable	Pregnancies		Abortions	
	**Pre-pandemic period** ^**a**^ **n (%)**	**Pandemic** **period** ^**b**^ **n (%)**	**Pre-pandemic period** ^**a**^ **n (%)**	**Pandemic** **period** ^**b**^ **n (%)**
	**n = 154,195**	**n = 78,399**	**n = 49,818**	**n = 36,911**
**Age, years**				
13–19	7,570 (4.9)	3,439 (4.4)	1,476 (3.0)	944 (2.6)
20–49	146,625 (95.1)	74,960 (95.6)	48,342 (97.0)	35,967 (97.4)
**Nationality**				
Georgian	106,507 (69.1)	68,483 (87.4)	30,534 (61.3)	31,839 (86.3)
Azerbaijani	10,789 (7.0)	5,879 (7.5)	2,691 (5.4)	2,765 (7.5)
Armenian	4,609 (3.0)	2,491 (3.2)	1,314 (2.6)	1,291 (3.5)
Other	3,154 (2.1)	1,546 (2.0)	1,281 (2.6)	1,017 (2.8)
Missing*	29,136 (18.9)	0	13,998 (28.1)	0
**Education**				
Primary	11,193 (7.3)	4,950 (6.3)	2,827 (5.7)	1,645 (4.5)
Secondary	68,228 (44.3)	34,227 (43.7)	24,750 (49.7)	18,248 (49.4)
Higher	44,215 (28.7)	21,062 (26.9)	8,041 (16.1)	4,930 (13.4)
Unknown*	30,559 (19.8)	18,160 (23.2)	14,200 (28.5)	12,088 (32.8)
**Residency**				
Urban	114,015 (74.0)	58,307 (74.4)	38,607 (77.5)	28,416 (77.0)
Rural	40,002 (26.0)	20,058 (25.6)	11,098 (22.3)	8,477 (23.0)
Missing*	178 (0.1)	34 (0.04)	113 (0.2)	18 (0.1)
**Method of abortion**				
Medical	N/A	N/A	20,203 (40.6)	16,943 (45.9)
Surgical	N/A	N/A	28,742 (57.7)	19,683 (53.3)
Missing*	N/A	N/A	873 (1.8)	285 (0.8)
**Gestational age at abortion, weeks (mean, SD)**	N/A	N/A	7.0 (2.9)	6.9 (2.8)

* Missing values refer to those entries that were either mistakenly or purposely not filled in, while “unknown” refers to entries where the category “unknown” has been selected.

^a^1 January 2018-31 March 2020; ^b^for pregnancies: 1 April 2020-30 June 2021; for abortions: 1 April 2020-30 April 2022.

SD: standard deviation.

When considering pregnancies or abortions among adolescents 13–19 years in the pre-pandemic period, most occurred in adolescents who were of Georgian nationality, had secondary education, and lived in urban areas (Table 2). Changes in the proportions of nationalities and education levels in the pandemic period are likely due to changes in the proportion of missing or unknown values. Compared to the pre-pandemic period, a slightly larger proportion (2.7% points) of abortions occurred in adolescents who lived in urban areas during the pandemic period.

Similar to the tendency among all women, we observed an increase in medical abortions (4.5% points) and a reduction in surgical abortions (-3% points) among adolescents 13–19 years in the pandemic period compared to the pre-pandemic period. Mean gestational age at abortion declined slightly from 7 weeks and 6 days to 7 weeks and 4 days.

**Table 2:** Maternal characteristics of pregnancies and abortions in adolescents (13–19 years)

Variable	Pregnancies		Abortions	
	**Pre-pandemic period** ^**a**^ **n (%)**	**Pandemic** **period** ^**b**^ **n (%)**	**Pre-pandemic period** ^**a**^ **n (%)**	**Pandemic** **period** ^**b**^ **n (%)**
	**n = 7,570**	**n = 3,439**	**n = 1,476**	**n = 944**
**Nationality**				
Georgian	4,146 (54.8)	2,191 (63.7)	823 (55.8)	703 (74.5)
Azerbaijani	2,106 (27.8)	1,108 (32.2)	234 (15.9)	192 (20.3)
Armenian	205 (2.7)	85 (2.5)	26 (1.8)	17 (1.8)
Other	137 (1.8)	55 (1.6)	50 (3.4)	32 (3.4)
Missing*	976 (12.9)	0	343 (23.2)	0
**Education**				
Primary	2,495 (33.0)	1,081 (31.4)	302 (20.5)	168 (17.8)
Secondary	3,279 (43.3)	1,405 (40.8)	676 (45.8)	415 (44.0)
Higher	440 (5.8)	187 (5.4)	82 (5.6)	53 (5.6)
Unknown*	1,356 (17.9)	766 (22.3)	416 (28.2)	308 (32.6)
**Residency**				
Urban	4,337 (57.3)	1,935 (56.3)	1040 (70.5)	691 (73.2)
Rural	3,223 (42.6)	1,503 (43.7)	428 (29.0)	253 (26.8)
Missing*	10 (0.1)	1 (0.03)	8 (0.5)	0
**Method of abortion**				
Medical	N/A	N/A	593 (40.2)	422 (44.7)
Surgical	N/A	N/A	843 (57.1)	511 (54.1)
Missing*	N/A	N/A	40 (2.7)	11 (1.2)
**Gestational age at abortion, weeks (mean, SD)**	N/A	N/A	7.9 (3.3)	7.6 (3.3)

* Missing values refer to those entries that were either mistakenly or purposely not completed, while “unknown” refers to entries where the category “unknown” was selected.

^a^1 January 2018-31 March 2020; ^b^for pregnancies: 1 April 2020-30 June 2021; for abortions: 1 April 2020-30 April 2022.

SD: standard deviation.

**Fig. 3:**
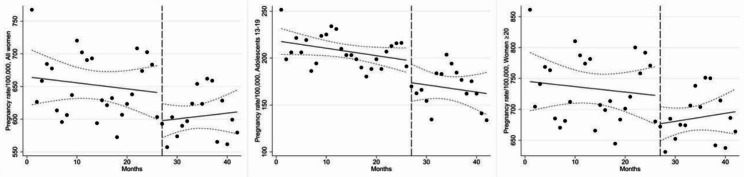
Pregnancy rates in all women (13–49 years); adolescents (13–19 years); and women (20–49 years). Pre-pandemic period: 1 January 2018-31 March 2020; Pandemic period: 1 April 2020-30 June 2021. Intervention started at month 27 which is indicated by the vertical dashed line

In January 2018, the pregnancy rate for all women was 664.0 per 100,000 women; for adolescents 13–19 years it was 217.7 and for women 20–49 years it was 744.8 per 100,000 women (Fig. 3; Table 3). During the pre-pandemic period, all age groups showed slightly declining trends in pregnancies (all women: -0.9 pregnancies per 100,000 women/month, 95% confidence interval (CI) -3.9 to 2.0; adolescents 13–19 years: -0.8 pregnancies per 100,000 adolescents 13–19 years/month, 95% CI -1.7 to 0.1; women 20–49 years: -0.9 pregnancies per 100,000 women 20–49 years/month, 95% CI -4.2 to 2.4).

During the 1-month strict lockdown, there was a drop in pregnancy rates in all investigated age groups, but it was most apparent in women 20–49 years, with a decrease of 45.1 (95% CI -103.2 to 13.0) pregnancies per 100,000 women 20–49 years. In adolescents 13–19 years, the pregnancy rate declined by 23.3 (95% CI -48.2 to 1.6) pregnancies per 100,000 adolescents 13–19 years that same month. During the pandemic period, there was an overall monthly increase of 0.9 (95% CI -2.4 to 4.2) pregnancies per 100,000 women; stratified by age, the monthly pregnancy rate among adolescents 13–19 years decreased by 0.8 (95% CI -3.2 to 1.6) pregnancies per 100,000 adolescents 13–19 years, while it increased in women 20–49 years by 1.3 (95% CI -2.2 to 4.8) pregnancies per 100,000 women 20–49 years. Moreover, compared to the pre-pandemic trend, the overall pandemic pregnancy trend declined more slowly (difference: 1.8 pregnancies per 100,000 women; 95% CI -2.6 to 6.2) and was mainly driven by women 20–49 years (difference: 2.2 pregnancies per 100,000 women; 95% CI -2.7 to 7.0). The single-group ITSAs per 5-year age group among women 20–49 years showed similar pregnancy trends to overall pregnancy tendencies in women 20–49 years, except for some minor differences (Supplementary Fig. 2 & Supplementary Table 2, Additional File 1). For instance, women 30–34, 35–39 and 40–49 years had an increasing monthly pregnancy rate change in the pre-pandemic period which was a contrast to the decreasing pregnancy trend in women 20–49 years. However, the pregnancy trends in women 20–24 years were more similar to the pregnancy trends in adolescents 13–19 years both in pre-pandemic and pandemic period, while the pregnancy tendencies among the rest of the 5-year age groups were more comparable to the patterns observed in women 20–49 years.

**Fig. 4:**
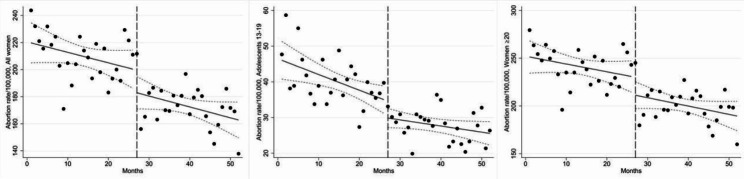
Abortion rates in all women (13–49 years); adolescents (13–19 years); and women (20–49 years). Pre-pandemic period:1 January 2018-31 March 2020; Pandemic period: 1 April 2020-30 April 2022. Intervention started at month 27 which is indicated by the vertical dashed line

The overall baseline abortion rate in all women in January 2018 was 219.8 per 100,000 women; 46.0 abortions per 100,000 adolescents 13–19 years and 251.2 abortions per 100,000 women 20–49 years. There was a decreasing trend in the pre-pandemic period in both age groups; it was most consistent for adolescents 13–19 years, with a reduction of 0.4 (95% CI -0.8 to -0.1) abortions per 100,000/month (Fig. 4; Table 3).

During the 1-month strict lockdown, there was a decrease in abortion rates in both age groups: -4.7 (95% CI -9.6 to 0.2) abortions per 100,000 adolescents 13–19 years and − 18.8 (95% CI -40.8 to 3.2) abortions per 100,000 women 20–49 years. The monthly abortion rate in the pandemic period also decreased in all age groups (all women: -0.8 abortions per 100,000 women, 95% CI -1.7 to 0.1; adolescents 13–19 years: -0.2 abortions per 100,000 adolescents 13–19 years, 95% CI -0.4 to 0.02; women 20–49 years: -0.9 abortions per 100,000 women 20–49 years, 95% CI -2.0 to 0.2). Compared to the pre-pandemic abortion trend, the pandemic trend appeared to decline slightly less in adolescents 13–19 years (difference: 0.3 abortions per 100,000 adolescents 13–19 years /month, 95% CI -0.1 to 0.7), whereas there was almost no difference in women 20–49 years (difference: -0.1 abortions per 100,000 women 20–49 years /month, 95% CI -1.7 to 1.6). Note that the CIs around the effect estimates indicated that both small increases and decreases in pregnancy and abortion rates are reasonably compatible with our data.

The single-group ITSAs per 5-year age group among women 20–49 years showed that the trends in abortion rates were comparable to the general abortion patterns in this age group, with only few small variations (Supplementary Fig. 3 & Supplementary Table 2, Additional File 1). Contrary to the overall abortion trend in women 20–49 years, women 20–25, 30–34 and 40–49 years had increasing abortion trends in the pandemic period compared to the pre-pandemic period. However, all the mentioned pregnancy and abortion tendencies in single-group ITSAs per 5-year age group had 95% CIs that included zero. Hence, the results should be interpreted with caution.

**Table 3:** Baseline rates, trends, and changes in pregnancy and abortion rates

	Baseline^a^ rate	Monthly rate change in the pre-pandemic period^b^	Immediate rate change during the 1-month strict lockdown^c^	Monthly rate change in the pandemic period^d^	Monthly rate difference in the pandemic period relative to the pre-pandemic period
	Pregnancies/abortions per 100,000 women	Pregnancies/abortions per 100,000/month (95% CI)	Pregnancies/abortions per 100,000 women (95% CI)	Pregnancies/abortions per 100,000/month (95% CI)	Pregnancies/abortions per 100,000/month (95% CI)
**Pregnancies**					
All women	664.0	-0.9 (-3.9 to 2.0)	-42.2 (-94.4 to 10.1)	0.9 (-2.4 to 4.2)	1.8 (-2.6 to 6.2)
Adolescents 13–19 years	217.7	-0.8 (-1.7 to 0.1)	-23.3 (-48.2 to 1.6)	-0.8 (-3.2 to 1.6)	0.03 (-2.5 to 2.6)
Women 20–49 years	744.8	-0.9 (-4.2 to 2.4)	-45.1 (-103.2 to 13.0)	1.3 (-2.2 to 4.8)	2.2 (-2.7 to 7.0)
**Abortions**					
All women	219.8	-0.8 (-1.8 to 0.3)	-16.7 (-35.5 to 2.1)	-0.8 (-1.7 to 0.1)	-0.03 (-1.4 to 1.4)
Adolescents 13–19 years	46.0	-0.4 (-0.8 to -0.1)	-4.7 (-9.6 to 0.2)	-0.2 (-0.4 to 0.02)	0.3 (-0.1 to 0.7)
Women 20–49 years	251.2	-0.8 (-2.0 to 0.4)	-18.8 (-40.8 to 3.2)	-0.9 (-2.0 to 0.2)	-0.1 (-1.7 to 1.6)

^a^January 2018; ^b^1 January 2018-31 March 2020; ^c^April 2020; ^d^for pregnancies: 1 April 2020-30 June 2021; for abortions: 1 April 2020-30 April 2022.

We compared changes in pregnancy and abortion rates in adolescents 13–19 years and women 20–49 years using the multiple ITSA approach (Supplementary Fig. 1 & Supplementary Table 1, Additional File 1). The results suggested that there were no substantial differences in changes in pregnancy and abortion rates in adolescents 13–19 years compared to women 20–49 years.

## Discussion

The COVID-19 pandemic and related restrictions had a great impact on the population of Georgia in terms of the country’s economy, access to education, and employment, especially in the younger generation. During the 1-month strict lockdown, we observed a decline in pregnancy and abortion rates in both adolescents 13–19 years and women 20–49 years, suggesting that this lockdown may have had an impact on access to pregnancy and abortion services and reproductive behavior/social contact in Georgia. However, this immediate effect was relatively small and short-lived, as we observed no substantial long-term impact on pregnancy or abortion rates during the study period. Our results, which were similar in adolescents 13–19 years and women 20–49 years, suggested that the various levels of COVID-19-related restrictions during the pandemic either (i) did not have a major impact on access to or utilization of SRH care services, or on women’s desire to have a child in Georgia; or (ii) that the changes in SRH care services and reproductive behavior were interrelated, and the combined effect resulted in no long-term changes in pregnancy and abortion rates. Additionally, given the fact that the number of babies born in Georgia has been declining since 2014 (Fig. 2) [25], it is unlikely that small changes in pregnancy and abortion rates during the pandemic will have a major impact on future demographics in Georgia.

Georgia experienced a steady decline in live births in the pre-pandemic period. The reasons for the decline are complex and may include economic distress, deterioration of social conditions, and emigration of women of childbearing age. There was a sharp increase in the number of live births in 2008 and 2014 (Fig. 2). The first rise was associated with an initiative from the leader of the Georgian Orthodox Church to baptize every child born to a family with at least two children; the second rise may have been associated with major changes in the country’s governing party. Nevertheless, these surges in live births were short-lived, and the birth rate declined from 2014 to 2021, when it returned to former levels.

Our findings of no substantial long-term changes in pregnancy or abortion rates in the pandemic compared to the pre-pandemic period are contrary to results from some previous studies that used data from the early phases of the pandemic, mostly performed in HICs, which suggested that the pandemic caused women to delay childbearing [26–29]. There was also speculation that the lack of availability of SRH care services in some countries, especially in LMICs, would lead to a massive surge of unplanned pregnancies and unsafe abortions [3, 5–7], which we did not observe in our study. Sobotka and colleagues [30] explored data from 22 HICs and reported a sharp decline in the monthly number of births when compared to the same month of the previous year in most of the countries, starting from October 2020. However, similar to our findings, no changes in births were observed in some countries, including Bulgaria, the Netherlands, Finland, Norway, and Slovenia. No country reported sustained increases in births [30]. Aassve et al. [13] also reported declining trends in crude birth rates in 18 out of 22 countries during the pandemic, but after adjustments these trends remained in only seven countries including Italy, Belgium, Spain, and Israel. In agreement with our study results, trends remained relatively stable in the rest of the countries [14].

In agreement with recommendations from the World Health Organization [31], Georgia prioritized access to healthcare for pregnant women from the very beginning of the pandemic [32]. The Ministry of Internally Displaced Persons from Occupied Territories, Labor, Health and Social Affairs of Georgia recommended that antenatal care providers use alternative remote service delivery, including phone and video conferencing. In addition to the use of several hotlines, media platforms, and social platforms for communication, Georgia quickly adopted remote consultations and support for pregnant women. Travel restrictions during the first lockdown did not negatively affect service delivery, as special movement permits allowed pregnant women to travel to service providers without obstacles, and such trips were generally shorter, given the lack of traffic [32]. These quick adaptations in antenatal care, as well as sustained delivery and postpartum care during the pandemic [32], may explain why we did not observe any sustained impact of COVID-19-related restrictions on pregnancy rates during the study period.

Our results suggested an increase in medical abortions and a decrease in surgical abortions in the pandemic period compared to the pre-pandemic period. These findings fit well with the fact that, even though some clinics discontinued surgical abortions, other medical facilities quickly adopted telemedicine services, including counselling, contraception, and post-abortion follow-up via phone or video conference [32]. Moreau et al. [33] reported that Georgia had already implemented a flexible system for dispensing medical abortion pills, with a similarly accommodating regulatory framework for home abortions before the pandemic. Additionally, a qualitative research project commissioned by the United Nations Population Fund studied the impact of COVID-19 social restrictions on access to SRH care services in Georgia in 2020; it suggested that there was a reduction in the supply of certain types of contraception in Georgia during the 1-month strict lockdown, except for contraceptive pills. Nevertheless, all pharmacies remained open and ensured access to all medicines, including contraception [32]. Additionally, in agreement with the immediate decline we observed in abortion rates, the United Nations Population Fund reported a decrease in abortions and limited access to abortion services during the 1-month strict lockdown [32]. However, these disruptions in abortion services may have been short-lived, as our results showed no substantial changes in abortion rates in the pre-pandemic and pandemic periods, and the mean gestational age at abortion remained relatively stable at around 7 weeks. Our findings may be attributed to the uninterrupted access to medical abortion pills through in-pharmacy and over-the-counter dispensing [33] which likely contributed to sustained access. This suggests that the SRH care services have not only demonstrated adaptability, but also sustainability.

### Strengths and limitations

One of the main strengths of the study is the large sample size and the use of a nationwide registry with 99% coverage of deliveries and newborns [21]. Another strength is the information completeness and validity of key variables in the study; date of birth and number of births are systematically validated against the Vital Registration System. These aspects make our study sample representative of the female Georgian population of reproductive age.

We used ITSA as our main analytical method, which assumes that no concurrent interventions are implemented at the same time as the intervention of interest. To our knowledge, no other intervention or change in policy took place during the study period. However, multiple points of intervention can be included in the ITSA model to better understand the impact of COVID-19-related restrictions on various reproductive rates. Due to overlapping restrictive policies during the pandemic, we were unable to include more intervention points in our model. We included the first, strict lockdown as a main intervention time-point and investigated the impact of all subsequent restrictions on abortion and pregnancy rates.

Some of the limitations of the present study include possible underreporting of abortions and our limited ability to differentiate between induced and spontaneous abortions. It appears reasonable to assume that an intervention such as a strict lockdown and the subsequent economic consequences of the pandemic may have had a larger influence on women’s desire to bear a child than on the risk of a miscarriage. Therefore, it would have been interesting to focus on induced abortions rather than total abortions. However, although induced abortion is legal in Georgia up to gestational week 13, and abortion due to social or medical reasons are legal up to week 22, it is still not socially accepted in various regions of the country. Induced abortion may therefore be underreported or misclassified as spontaneous abortions; Thus, we chose to investigate total abortions in this study.

A limitation of the study is that the values in the nationality variable may not have been missing completely at random before 2019, when this variable became mandatory. However, we do not think this had a major impact on our results since the distribution of nationalities was similar to the pandemic period.

## Conclusions

Our results suggest that the 1-year-long COVID-19-related restrictions in Georgia had no substantial long-term influence on pregnancy and abortions rates during the study period among adolescents 13–19 years or women 20–49 years. Consequently, it is possible that the restrictions did not impact access to SRH care services and contraception, or reproductive behavior; or that simultaneous changes occurred in all mentioned factors that cancelled out the total long-term effect.

### Electronic supplementary material

Below is the link to the electronic supplementary material.

Supplementary Material 1: **Additional File 1**: GBR data on pregnancies and abortions. Description of data: Results of multiple interrupted time series analysis (ITSA) comparing pregnancy and abortion rates in pre-pandemic and pandemic periods in adolescents (13–19 years) and women (20–49 years), and single-group ITSAs per 5-year age groups among women 20–49 years.

## Data Availability

The data that support the findings of this study are available from the National Center for Disease Control and Public Health of Georgia, but restrictions apply to the availability of these data, which were used under license for the current study, and so are not publicly available. Data are however available from the corresponding author upon reasonable request and with permission of the National Center for Disease Control and Public Health of Georgia. For permission you can contact National Center for Disease Control and Public Health of Georgia via pr@ncdc.ge.

## List of abbreviations

CI: Confidence interval
GBR: Georgian Birth Registry
HICs: High-income countries
IRB: Institutional Review Board
ITSA: Interrupted time series analysis
LMICs: Low- and middle-income countries
SRH: Sexual and reproductive health
