# Supplementary material for: The impact of COVID-19-related restrictions on pregnancy and abortion rates in the Republic of Georgia
Source: BMC Health Serv Res. 2023 Dec 18;23:1435. doi: 10.1186/s12913-023-10417-7 (PMC10729579; doi:10.1186/s12913-023-10417-7)
Supplement: Supplementary file 1 — Supplementary Material 1: Additional File 1: GBR data on pregnancies and abortions. Description of data: Results of multiple interrupted time series analysis (ITSA) comparing pregnancy and abortion rates in pre-pandemic and pandemic periods in adolescents (13–19 years) and women (20–49 years), and single-group ITSAs per 5-year age groups among women 20–49 years. [file 12913_2023_10417_MOESM1_ESM.docx]

# **The impact of COVID-19-related restrictions on pregnancy and abortion rates in the Republic of Georgia**

**BMC Health Services Research**

**Nia Khachidze^1*^, Tinatin Manjavidze^1^, Erik Eik Anda^1^, Ingvild Hersoug Nedberg^2^, Ingvild Fossgard Sandøy^3^, Charlotta Rylander^1^**

^1^Department of Community Medicine, Faculty of Health Sciences, UiT The Arctic University of Norway, Tromsø, Norway

^2^Department of Health and Care Sciences, Faculty of Health Sciences, UiT The Arctic University of Norway, Tromsø, Norway

^3^Center for Intervention Science in Maternal and Child Health, Centre for International Health, Department of Global Public Health and Primary Care, University of Bergen, Bergen, Norway

*Corresponding author, [nia.khachidze@uit.no](mailto:nia.khachidze@uit.no)

# Supplementary Information

**Supplementary Fig. 1** Multiple ITSA comparing pregnancy and abortion rates in adolescents (13-19 years) and women (20-49 years).

**
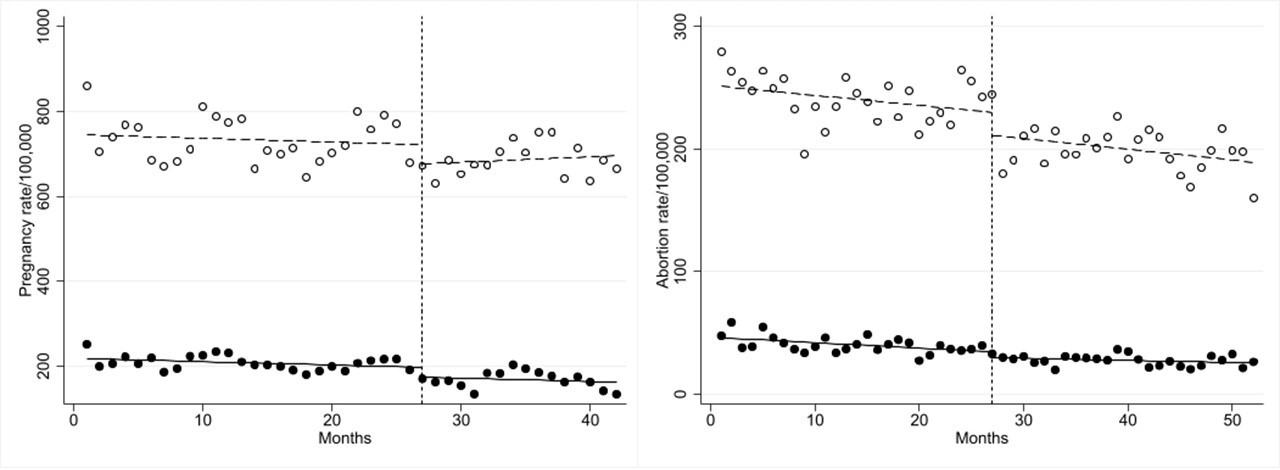
**

Pre-pandemic period: 1 January 2018-31 March 2020; Pandemic period for pregnancies: 1 April 2020-30 June 2021; for abortions: 1 April 2020-30 April 2022. Adolescents: 13-19 years (• - treated); women: 20-49 years (○ - controls). Intervention started at month 27.

ITSA: Interrupted time series analysis

**Supplementary Table 1** Baseline rates, trends, and changes in pregnancy and abortion rates in adolescents and women 20-49 years

|  | Baseline rate^a^ in older women | Monthly rate change in older women in the pre-pandemic period^b^ | Baseline rate^a^ difference in adolescents compared to older women | Rate difference in adolescents compared to older women in the pre-pandemic period^b^ | Immediate rate change during the 1- month strict lockdown^c^ in adolescents | Monthly rate difference in older women in the pandemic period^d^ compared to the pre-pandemic period^c^ | Additional rate change in adolescents compared to older women during the 1-month strict lockdown^c^ | Additional rate difference in the pandemic period^d^ compared to the pre-pandemic period^c^ in adolescents compared to older women |
| --- | --- | --- | --- | --- | --- | --- | --- | --- |
|  | Pregnancies/abortions per 100,000 women | Pregnancies/abortions per 100,000/month (95% CI) | Pregnancies/abortions per 100,000 women (95% CI) | Pregnancies/abortions per 100,000 women (95% CI) | Pregnancies/abortions per 100,000 women (95% CI) | Pregnancies/abortions per 100,000/month (95% CI) | Pregnancies/abortions per 100,000 women (95% CI) | Pregnancies/abortions per 100,000/month (95% CI) |
| Pregnancies | 744.8 | -0.9 (-4.2 to 2.4) | -527.2 | -0.1 (-3.3 to 3.5) | -45.1 (-102.2 to 12.1) | 2.19 (-2.5 to 7.0) | 21.8 (-40.4 to 84.0) | -2.16 (-7.6 to 3.2) |
| Abortions | 251.2 | -0.8 (-2.0 to 0.4) | -205.2 | 0.4 (-0.9 to 1.6) | -18.8 (-40.5 to 2.9) | -0.1 (-1.7 to 1.6) | 14.1 (-8.16 to 36.4) | 0.33 (-1.3 to 2.0) |

^a^January 2018; ^b^1 January 2018-31 March 2020; ^c^April 2020; ^d^for pregnancies: 1 April 2020-30 June 2021; for abortions: 1 April 2020-30 April 2022. Adolescents: 13-19 years; women: 20-49 years.

CI: confidence interval

**Supplementary Fig. 2** Pregnancy rates in women aged 20-24, 25-29, 30-34, 35-39, 40-49 years.

**
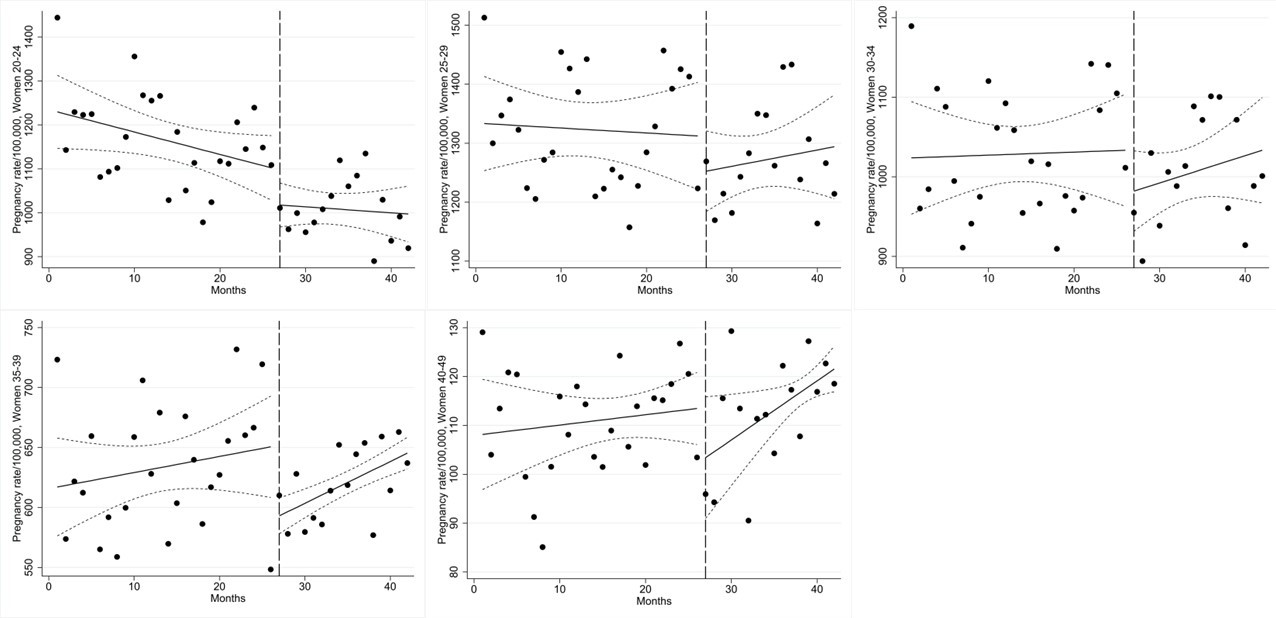
**

Pre-pandemic period: 1 January 2018-31 March 2020; Pandemic period: 1 April 2020-30 June 2021. Intervention started at month 27 which is indicated by the vertical dashed line.

**Supplementary Fig. 3** Abortion rates in women aged 20-24, 25-29, 30-34, 35-39, 40-49 years.


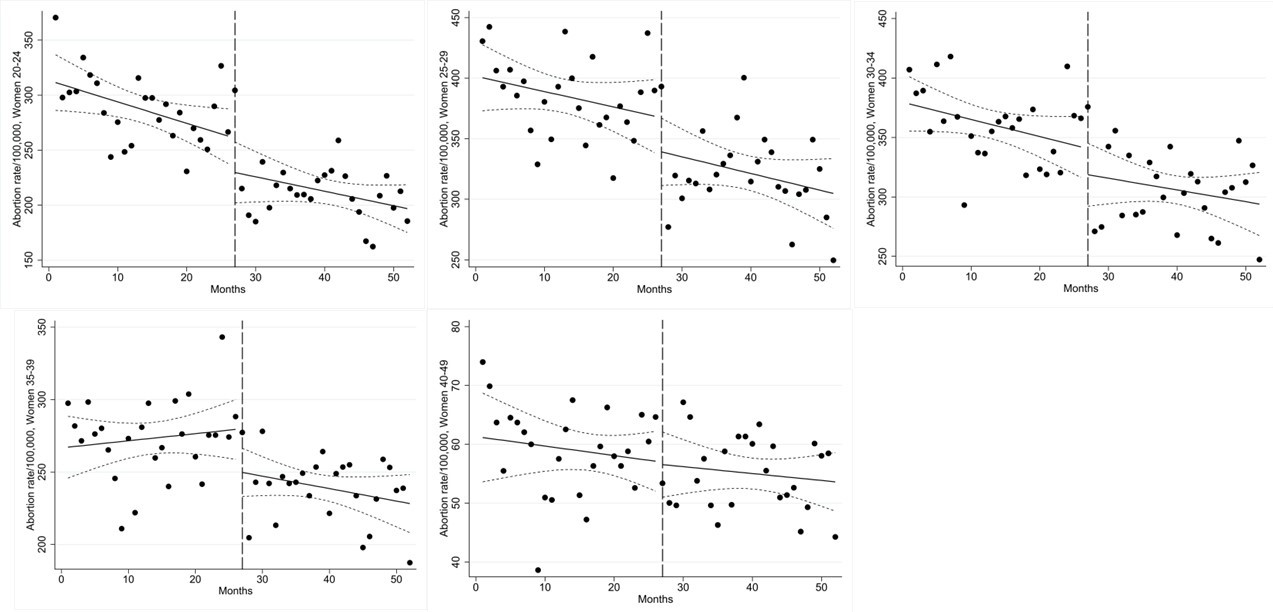


Pre-pandemic period: 1 January 2018-31 March 2020; Pandemic period: 1 April 2020-30 June 2021. Intervention started at month 27 which is indicated by the vertical dashed line.

**Supplementary Table 2** Baseline rates, trends, and changes in pregnancy and abortion rates

|  | Baseline^a^ rate | Monthly rate change in the pre-pandemic period^b^ | Immediate rate change during the 1-month strict lockdown^c^ | Monthly rate change in the pandemic period^d^ | Monthly rate difference in the pandemic period relative to the pre-pandemic period |
| --- | --- | --- | --- | --- | --- |
|  | Pregnancies/abortions per 100,000 women | Pregnancies/abortions per 100,000/month (95% CI) | Pregnancies/abortions per 100,000 women (95% CI) | Pregnancies/abortions per 100,000/month (95% CI) | Pregnancies/abortions per 100,000/month (95% CI) |
| **Pregnancies** | | | | | |
| Women 20-24 | 1229.8 | -5.1 (-10.5 to 0.3) | -79.5 (-175.1 to 16.0) | -1.4 (-7.4 to 4.7) | 3.7 (-4.4 to 11.9) |
| Women 25-29 | 1333.2 | -0.8 (-6.8 to 5.1) | -58.9 (-181.4 to 63.7.6) | 2.8 (-6.0 to 11.5) | 3.6 (-6.9 to 14.1) |
| Women 30-34 | 1023.8 | 0.4 (-4.7 to 5.5) | -51.8 (-144.5 to 41.0) | 3.4 (-3.3 to 10.1) | 3.0 (-5.4 to 11.5) |
| Women 35-39 | 617.0 | 1.3 (-1.7 to 4.4) | -58.9 (-110.5 to 7.4) | 3.5 (2.0 to 4.9) | 2.1 (-0.9 to 5.2) |
| Women 40-49 | 108.2 | 0.2 (-0.5 to 0.9) | -10.3 (-25.1 to 4.6) | 1.2 (0.1 to 2.3) | 0.9 (-0.3 to 2.3) |
| **Abortions** | | | | | |
| Women 20-24 | 311.4 | -2.0 (-3.7 to -0.1) | -31.0 (-69.9 to 7.8) | -1.3 (-3.1 to 0.5) | 0.6 (-1.9 to 3.2) |
| Women 25-29 | 400.4 | -1.3 (-3.3 to 0.8) | -28.2 (-70.4 to 13.9) | -1.4 (-3.4 to 0.6) | -0.1 (-3.1 to 2.8) |
| Women 30-34 | 378.2 | -1.5 (-3.2 to 0.3) | -21.9 (-59.1 to 15.2) | -1.0 (-2.9 to 1.0) | 0.5 (-2.2 to 3.2) |
| Women 35-39 | 267.1 | 0.5 (-1.0 to 2.0) | -30.1 (-57.9 to -2.4) | -0.9 (-2.2 to 0.5) | -1.4 (-3.4 to 0.7) |
| Women 40-49 | 61.2 | -0.2 (-0.6 to 0.3) | -0.4 (-8.5 to 7.7) | -0.1 (-0.5 to 0.3) | 0.04 (-0.5 to 0.6) |

^a^January 2018; ^b^1 January 2018-31 March 2020; ^c^April 2020; ^d^for pregnancies: 1 April 2020-30 June 2021; for abortions: 1 April 2020-30 April 2022.
